# Liver Anatomy Quiz: Test Your Knowledge

**DOI:** 10.1007/s11605-020-04582-x

**Published:** 2020-05-14

**Authors:** David A. Geller, Samer Tohme

**Affiliations:** Division of Hepatobiliary and Pancreatic Surgery, Department of Surgery, University of Pittsburgh, MUH 7 South, Pittsburgh, PA 15213 USA

## Abstract

Understanding liver anatomy and anatomic hilar vascular variants is important for the practicing surgeon. This knowledge is essential for cholecystectomy, hepatobiliary, pancreatic, and upper GI surgery. The attached quiz is intended to provide a liver anatomy teaching guide for surgical oncology, transplant, and HPB fellows; general surgery residents; and medical students, as well as a refresher for general and GI surgeons. It is hoped that dissemination will serve as a valuable teaching tool for surgeons at all levels of training.

## Liver Anatomy Exam: 75 Questions, Multiple Choice/Matching

1. Liver segmental anatomy is named after which physician that defined the hepatic segments in the 1950s:

A. Giulio Arantius
B. J Cantlie
C. Claude Couinaud
D. Thomas Starzl
E. Henri Bismuth

2. Current liver resection terminology was coined at which IHPBA meeting/consensus?

A. 1998 Madrid
B. 2000 Brisbane
C. 2002 Tokyo
D. 2004 Washington D.C.
E. 2006 Edinburgh

3. The liver is divided into 2 lobes or hemi-livers.

A. True
B. False

4. A replaced right hepatic artery originates from which structure?

A. Aorta
B. Superior mesenteric artery
C. Celiac axis
D. Splenic artery
E. Left gastric artery

5. A replaced left hepatic artery originates from which structure?

A. Aorta
B. Superior mesenteric artery
C. Celiac axis
D. Gastroduodenal artery
E. Left gastric artery

6. Venous drainage of the hepatic segments is through which hepatic veins?

A. Right, middle, left, and short hepatic veins
B. Right, left, and center hepatic veins
C. Right, left, and short hepatic veins
D. Right, left, and long hepatic veins
E. Right, middle, left, and long hepatic veins

7. The right lobe of the liver includes which segments?

A. 2, 3, 4
B. 1, 2, 3, 4
C. 5, 6, 7, 8
D. 3, 4, 5, 6
E. 1, 2, 7, 8

8. The left lobe liver includes which segments?

A. 2, 3, 4
B. 1, 2, 3, 4
C. 5, 6, 7, 8
D. 3, 4, 5, 6
E. 1, 2, 7, 8

9. Regarding segmental hepatic anatomy, which of the following is the most accurate statement?

A. The right lobe is divided into medial and lateral segments.
B. The right lobe is divided into anterior and posterior segments.
C. The left lobe is divided into medial and lateral segments.
D. The left lobe is divided into anterior and posterior segments.
E. Both A and D are true.
F. Both B and C are true.

10. The venous drainage of the caudate lobe is into which structure?

A. Left hepatic vein
B. Middle hepatic vein
C. Right hepatic vein
D. Inferior vena cava
E. Portal vein

11. For hepatic lobar anatomy, which of the following is correct?

A. The falciform ligament separates the right and left lobes of the liver.
B. The plane from the gallbladder bed to the IVC (Cantlie’s line) separates the right and left lobes.
C. The falciform ligament separates the left lateral and left medial segments.
D. Both A and B are correct.
E. Both B and C are correct.

12. The portal vein is formed from confluence of which veins?

A. Splenic vein and inferior mesenteric vein
B. Splenic vein and superior mesenteric vein
C. Splenic vein and gastric vein
D. Superior mesenteric vein and inferior mesenteric vein
E. None of the above

13. With severe portal HTN, portal blood flow is often:

A. Hepatofugal.
B. Hepatopetal.
C. Diverted through the coronary vein.
D. Both A and C are correct.
E. Both B and C are correct.

14. The left medial hepatic segment is also known as:

A. Segment IV
B. Quadrate lobe
C. Caudate lobe
D. Both A and C
E. Both A and B

15. A replaced right hepatic artery typically courses:

A. Posterior to the portal vein
B. Anterior to the portal vein
C. In the gastrohepatic ligament
D. Both A and C are correct
E. Both B and C are correct

16. Conventional or normal hepatic artery blood flow is which of the following:

A. Aorta to celiac axis to hepatic artery proper to common hepatic artery to R/L hepatic arteries
B. Aorta to celiac axis to common hepatic artery to hepatic artery proper to R/L hepatic arteries
C. Aorta to common hepatic artery to celiac axis to hepatic artery proper to R/L hepatic arteries
D. None of the above

17. A central liver resection for gallbladder cancer typically involves removing all or a portion of which hepatic segments?

A. 5 and 4B
B. 5 and 4A
C. 5 and 6
D. 5 and 8
E. 6 and 7

18. A right hepatic trisectionectomy (or trisegmentectomy) removes which segments?

A. 1, 2, 3, 4, 5
B. 2, 3, 4, 5, 6
C. 2, 3, 4, 5, 8
D. 1, 5, 6, 7, 8
E. 4, 5, 6, 7, 8

19. A left hepatic trisectionectomy (or trisegmentectomy) removes which segments?

A. 1, 2, 3, 4, 7
B. 2, 3, 4, 5, 8
C. 1, 2, 3, 4, 6, 7
D. Both A and B are correct.
E. Both B and C are correct.

20. The liver has how many segments?

A. 5
B. 6
C. 7
D. 8
E. 10

21. Arantius’ ligament is the:

A. Ligamentum venosum
B. Obliterated ductus venosus
C. Obliterated hepatic vein
D. Both A and B
E. Both A and C

22. Aberrant biliary anatomy with the right anterior or posterior hepatic duct draining into left hepatic duct occurs what percent of time?

A. 5%
B. 10%
C. 25%
D. 60%
E. 80%

23. The fissure of Gans:

A. Is embryologically present 70% of time
B. Contains the right posterior sectoral pedicle
C. Is also referred to as Rouviere’s sulcus
D. All of the above
E. None of the above

24. The right hepatic artery crosses:

A. Posterior to the common hepatic duct 88% of time and anterior to common hepatic duct 12% of time
B. Posterior to the common hepatic duct 12% of time and anterior to common hepatic duct 88% of time
C. Posterior to the common hepatic duct 50% of the time
D. Posterior to the common hepatic duct 100% of the time
E. None of the above

25. The left and middle hepatic veins form a common trunk before inserting into the supra-hepatic IVC in what % of time?

A. 20%
B. 40%
C. 60%
D. 80%
E. 95%

26. The segment 4 portal vein branches originate from which vessel?

A. Right PV
B. Left PV
C. Main PV
D. Caudate vein
E. None of the above

27–35. Match the hepatic segments on the CT scan images:

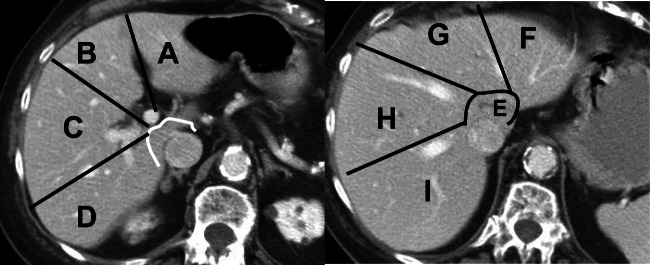



Segment

27. 1 ___________

28. 2 ___________

29. 3 ___________

30. 4A __________

31. 4B __________

32. 5 ___________

33. 6 ___________

34. 7 ___________

35. 8 ___________

36–39. Match the hepatic structures on the CT image:

36. IVC __________

37. RHV _________

38. MHV ________

39. LHV _________

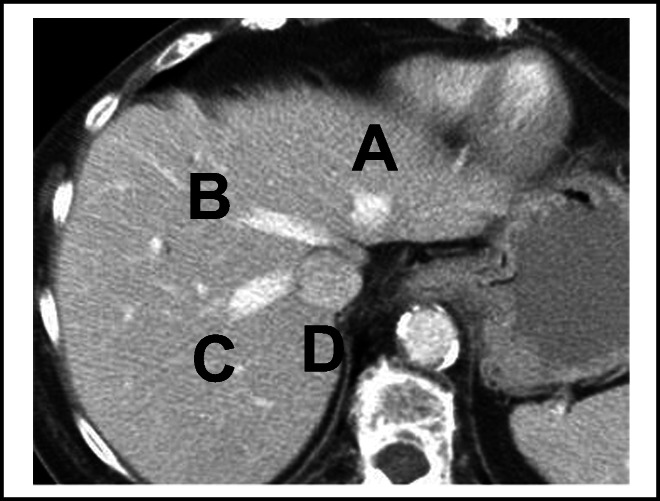



40–45. Match the hepatic structures on the US images:

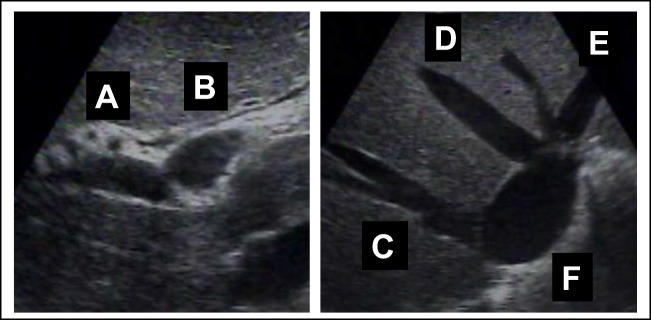



40. LPV ______________________

41.RPV ______________________

42. IVC ______________________

43. RHV _____________________

44. MHV_____________________

45.LHV ______________________

46–48. Label the hilar structures on the ultrasound:

46. Common hepatic duct ______________

47. HA ______________

48. PV ______________

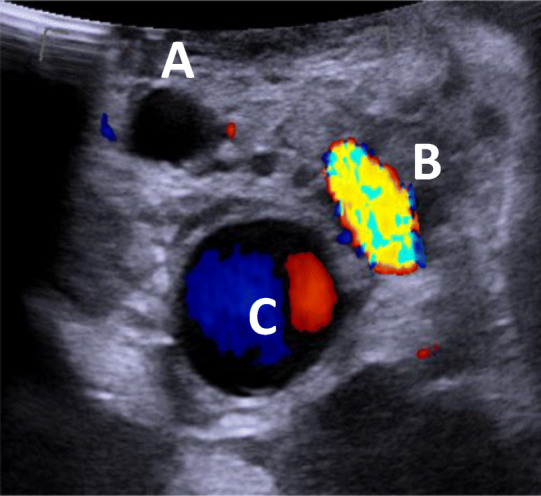



49. In a healthy adult, what is the minimum percent of future liver remnant typically needed to allow for extended lobectomy or trisectionectomy?

A. 90%

B. 70%

C. 50%

D. 25%

E. 10%

50. The hepatoduodenal ligament contains which structures?

A. Portal vein
B. Hepatic artery
C. Common Bile duct
D. All of the above
E. A and B only

51. The space passing behind the hepatoduodenal ligament to enter the lesser sac is known as:

A. Epiploic foramen
B. Duodenal tunnel
C. Foramen of Winslow
D. Both A and B
E. Both A and C

52. The round ligament is also known as:

A. Ligamentum teres
B. Arantius ligament
C. Ductus venosum
D. Left triangular ligament
E. Falciform ligament

53. The cystic artery most commonly arises from the:

A. Hepatic artery proper
B. Common hepatic artery
C. Right hepatic artery
D. Left hepatic artery
E. Gastroduodenal artery

54. Suspensory ligaments of the liver include:

A. Falciform ligament
B. Left triangular ligament
C. Right triangular ligament
D. Round ligament
E. All of the above

55. The left lateral segment/section is made up of which hepatic segments?

A. 1 and 2
B. 2 and 3
C. 3 and 4
D. 4 and 5
E. 5 and 8

56. When placing a hepatic artery infusion (HAI) pump, the tip of the catheter tubing should be in the:

A. Right hepatic artery
B. Hepatic artery proper
C. Common hepatic artery
D. Gastroduodenal artery
E. Celiac axis

57. Surgical strategies to enhance operability of liver tumors include:

A. Right portal vein embolization
B. ALPPS
C. Two-stage hepatectomy
D. Combining liver resection with ablation
E. All of the above

58. In Budd-Chiari syndrome:

A. The portal veins are thrombosed.
B. The hepatic veins are thrombosed.
C. Ascites is rarely present.
D. A hypercoagulable work-up should be done.
E. Both B and D are correct.

59. When considering future liver remnant for hepatic trisectionectomy:

A. 20–25% future liver remnant is usually adequate for normal liver.
B. > 30% future liver remnant is preferred in fatty liver.
C. > 40% future liver remnant is recommended for severe fibrosis or cirrhotic liver.
D. ICG clearance is helpful for cirrhotic livers.
E. All of the above.

60. Gilbert’s disease:

A. Is a hyperbilirubinemia with serum T bili usually > 5
B. Is a progressive disease that usually leads to liver failure
C. Requires treatment with plasmapheresis
D. Is an autosomal recessive disease with mildly elevated levels of unconjugated bilirubin and normally no serious consequences
E. All of the above

61–75. Match the letter to the correct liver segment: 1, 2, 3, 4A, 4B, 5, 6, 7, 8.

(A segment may be used more than once)

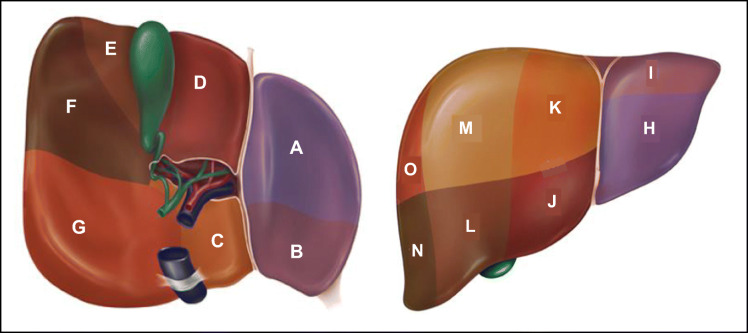



Segment #

61. A ___________

62. B. __________

63. C. __________

64. D. __________

65.E. ___________

66. F. __________

67. G.___________

68. H.___________

69. I.___________

70. J.__________

71. K.___________

72. L.____________

73. M. ___________

74. N.___________

75. O.___________

### Answer Key: Liver Anatomy Exam (Correct Answers Are Highlighted) 1–6

1. Liver segmental anatomy is named after which physician that defined the hepatic segments in the 1950s:

A. Giulio Arantius
B. J Cantlie
C. **Claude Couinaud**
D. Thomas Starz
E. Henri Bismuth

Claude Couinaud published his classic description of liver anatomy in 1954 in the French literature. Couinaud C, Lobes de segments hepatiques: Notes sur l’architecture anatomique et chirurgical de foie, *Presse Méd*. 1954; 62:709–715. Giulio Arantius was an Italian anatomist who made many contributions to human anatomy, fetal circulation, and science. James Cantlie was a Scottish surgeon who described the midline of the liver between the right and left lobes based on autopsy findings in 1887. This line passes from the gallbladder fossa down to the inferior vena cava. Thomas Starzl was a pioneer in the field of organ transplantation and performed the world’s first liver transplant in 1963 in Denver, CO. Henri Bismuth is a French pioneer in hepatobiliary surgery and is credited with developing the split-liver technique for liver transplantation that allows two patients to be transplanted from only one liver donor.

2. Current liver resection terminology was coined at which IHPBA meeting/consensus?

A. 1998 Madrid
B. **2000 Brisbane**
C. 2002 Tokyo
D. 2004 Washington D.C.
E. 2006 Edinburgh

Confusion existed in the literature regarding liver resection terminology. The Brisbane 2000 Nomenclature of Hepatic Anatomy and Resections was established to standardize liver resection terminology.

3. The liver is divided into 2 lobes or hemi-livers

A. **True**
B. False

4. A replaced right hepatic artery originates from which structure?

A. Aorta
B. **Superior mesenteric artery**
C. Celiac axis
D. Splenic artery
E. Left gastric artery

5. A replaced left hepatic artery originates from which structure?

A. Aorta
B. Superior mesenteric artery
C. Celiac axis
D. Gastroduodenal artery
E. **Left gastric artery**

6. Venous drainage of the hepatic segments are through which hepatic veins;

A. **Right, middle, left, and short hepatic veins**
B. Right, left, and center hepatic veins
C. Right, left, and short hepatic veins
D. Right, left, and long hepatic veins.
E. Right, middle, left, and long hepatic veins.

7. The right lobe of the liver includes which segments?

A. 2, 3, 4
B. 1, 2, 3, 4
C. **5, 6, 7, 8**
D. 3, 4, 5, 6
E. 1, 2, 7, 8

8. The left lobe liver includes which segments?

A. **2, 3, 4**
B. 1, 2, 3, 4
C. 5, 6, 7, 8
D. 3,4,5,6
E. 1,2,7,8

9. Regarding segmental hepatic anatomy, which of the following is the most accurate statement?

A. The right lobe is divided into medial and lateral segments.
B. The right lobe is divided into anterior and posterior segments.
C. The left lobe is divided into medial and lateral segments.
D. The left lobe is divided into anterior and posterior segments.
E. Both A and D are true
F. **Both B and C are true.**

10. The venous drainage of the caudate lobe is into which structure?

A. Left hepatic vein
B. Middle hepatic vein
C. Right hepatic vein
D. **Inferior vena cava**
E. Portal vein

11. For hepatic lobar anatomy, which of the following is correct?

A. The falciform ligament separates the right and left lobes of the liver.
B. The plane from the gallbladder bed to the IVC (Cantlie’s line) separates the right and left lobes.
C. The falciform ligament separates the left lateral and left medial segments.
D. Both A and B are correct.
E. **Both B and C are correct.**

12. The portal vein is formed from confluence of which veins?

A. Splenic vein and inferior mesenteric vein
B. **Splenic vein and superior mesenteric vein**
C. Splenic vein and gastric vein
D. Superior mesenteric vein and inferior mesenteric vein
E. None of the above

13. With severe portal HTN, portal blood flow is often:

A. Hepatofugal.
B. Hepatopetal.
C. Diverted through the coronary vein.
D. **Both A and C are correct.**
E. Both B and C are correct.

Normal blood flow is from the portal vein through the liver (hepatopetal flow). In the setting of cirrhosis and portal hypertension, blood flow reverses away from the liver (hepatofugal flow) and often passes through the dilated coronary vein and/or re-canalized umbilical vein.

14. The left medial hepatic segment is also known as:

A. Segment IV
B. Quadrate lobe
C. Caudate lobe
D. Both A and C
E. **Both A and B**

The left medial segment of the liver is also known as segment IV or the quadrate lobe of the liver. Hence, it has 3 names that refer to the same segment.

15. A replaced right hepatic artery typically courses;

A. **Posterior to the portal vein**
B. Anterior to the portal vein
C. In the gastrohepatic ligament
D. Both A and C are correct
E. Both B and C are correct

16. Conventional or normal hepatic artery blood flow is which of the following:

A. Aorta to celiac axis to hepatic artery proper to common hepatic artery to R/L hepatic arteries
B. **Aorta to celiac axis to common hepatic artery to hepatic artery proper to R/L hepatic arteries**
C. Aorta to common hepatic artery to celiac axis to hepatic artery proper to R/L hepatic arteries
D. None of the above

17. A central liver resection for gallbladder cancer typically involves removing all or a portion of which hepatic segments?

A. **5 and 4B**
B. 5 and 4A
C. 5 and 6
D. 5 and 8
E. 6 and 7

18. A right hepatic trisectionectomy (or trisegmentectomy) removes which segments?

A. 1, 2, 3, 4, 5
B. 2, 3, 4, 5, 6
C. 2, 3, 4, 5, 8
D. 1, 5, 6, 7, 8
E. **4, 5, 6, 7, 8**

19. A left hepatic trisectionectomy (or trisegmentectomy) removes which segments?

A. 1, 2, 3, 4, 7
B. **2, 3, 4, 5, 8**
C. 1, 2, 3, 4, 6, 7
D. Both A and B are correct
E. Both B and C are correct.

20. The liver has how many segments?

A. 5
B. 6
C. 7
D. **8**
E. 10

Liver segmental anatomy was described by Claude Couinaud in 1954.

21. Arantius’ ligament is the:

A. Ligamentum venosum
B. Obliterated ductus venosus
C. Obliterated hepatic vein
D. **Both A and B**
E. Both A and C

22. Aberrant biliary anatomy with the right anterior or posterior hepatic duct draining into left hepatic duct occurs what percent of time?

A. 5%
B. 10%
C. **25%**
D. 60%
E. 80%

23. The fissure of Gans:

A. Is embryologically present 70% of time
B. Contains the right posterior sectoral pedicle
C. Is also referred to as Rouviere’s sulcus
D. **All of the above**
E. None of the above

24. The right hepatic artery crosses:

A. **Posterior to the common hepatic duct 88% of time and anterior to common hepatic duct 12% of time**
B. Posterior to the common hepatic duct 12% of time and anterior to common hepatic duct 88% of time
C. Posterior to the common hepatic duct 50% of the time
D. Posterior to the common hepatic duct 100% of the time
E. None of the above

25. The left and middle hepatic veins form a common trunk before inserting into the supra-hepatic IVC in what % of time?

A. 20%
B. 40%
C. 60%
D. 80%
E. **95%**

26. The segment 4 portal vein branches originate from which vessel?

A. Right PV
B. **Left PV**
C. Main PV
D. Caudate vein
E. None of the above

27–35. Match the hepatic segments on the CT scan images:
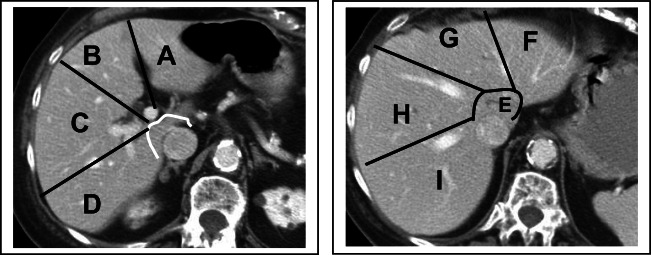


Segment

27.1 _____**E**_______

28.2 _____**F**_______

29.3 _____**A**_______

30.4A _____**G**______

31.4B ____**B**_______

32. 5 _____**C**_______

33. 6 **_____D**______

34. 7 **_____I**_______

35. 8 _____**H**______

36–39. Match the hepatic structures on the CT image:
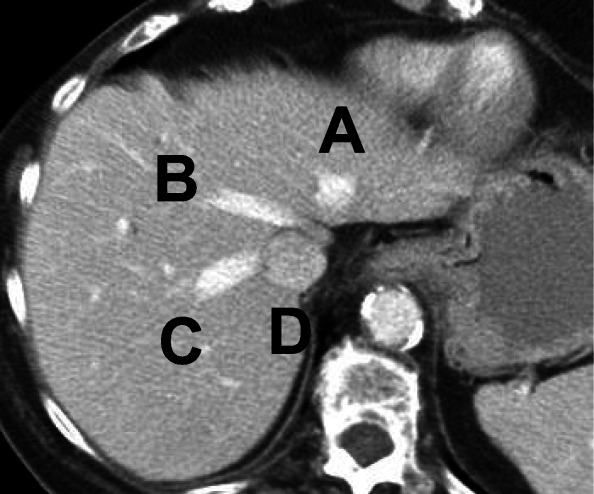


36. IVC ___**D**_______

37. RHV ___**C**______

38. MHV ___**B**_____

39. LHV ___**A_**_____

40–45. Match the hepatic structures on the US images:
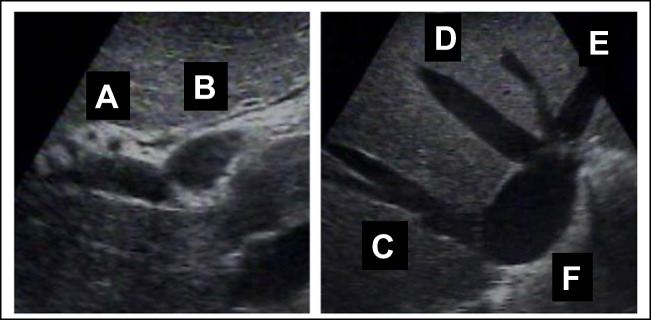


40. LPV ______**B**_____________

41.RPV ______**A_**____________

42. IVC **______F**_____________

43. RHV ______**C_**___________

44. MHV ______**D___________**

45.LHV **______E_____________**

46–48. Label the hilar structures on the ultrasound:

46. Common hepatic duct **______A________**

47. HA **______B**________

48. PV **______C________**
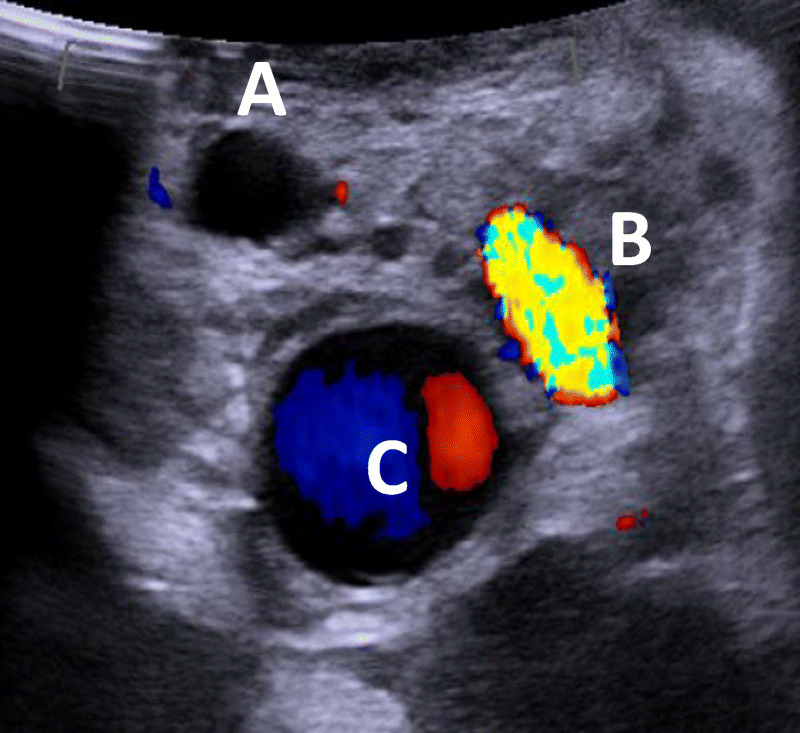


49. In a healthy adult, what is the minimum percent of future liver remnant typically needed to allow for extended lobectomy or trisectionectomy?

A. 90%
B. 70%
C. 50%
D. **25%**
E. 10%

50. The hepatoduodenal ligament contains which structures?

A. Portal vein
B. Hepatic artery
C. Common Bile duct
D. **All of the above**
E. A and B only

51. The space passing behind the hepatoduodenal ligament to enter the lesser sac is known as:

A. Epiploic foramen
B. Duodenal tunnel
C. Foramen of Winslow
D. Both A and B
E. **Both A and C**

52. The round ligament is also known as:

A. **Ligamentum teres**
B. Arantius ligament
C. Ductus venosum
D. Left triangular ligament
E. Falciform ligament

53. The cystic artery most commonly arises from the:

A. Hepatic artery proper
B. Common hepatic artery
C. **Right hepatic artery**
D. Left hepatic artery
E. Gastroduodenal artery

54. Suspensory ligaments of the liver include:

A. Falciform ligament
B. Left triangular ligament
C. Right triangular ligament
D. Round ligament
E. **All of the above**

55. The left lateral segment/section is made up of which hepatic segments?

A. 1 and 2
B. **2 and 3**
C. 3 and 4
D. 4 and 5
E. 5 and 8

56. When placing a hepatic artery infusion (HAI) pump, the tip of the catheter tubing should be in the:

A. Right hepatic artery
B. Hepatic artery proper
C. Common hepatic artery
D. **Gastroduodenal artery**
E. Celiac axis

When placing a hepatic artery infusion (HAI) pump, the tip of the catheter tubing should be in the gastroduodenal artery just prior to the hepatic artery. The goal is to perfuse the entire liver via the hepatic artery proper and right/left hepatic artery branches. A 5-cm gastro-duodenal devascularization should be done to avoid mal-perfusion of chemotherapy to the duodenum which can lead to ulceration/bleeding.

57. Surgical strategies to enhance operability of liver tumors include:

A. Right portal vein embolization
B. ALPPS
C. Two-stage hepatectomy
D. Combining liver resection with ablation
E. **All of the above**

58. In Budd-Chiari syndrome:

A. The portal veins are thrombosed.
B. The hepatic veins are thrombosed.
C. Ascites is rarely present.
D. A hypercoagulable work-up should be done.
E. **Both B and D are correct.**

59. When considering future liver remnant for hepatic trisectionectomy:

A. 20–25% future liver remnant is usually adequate for normal liver.
B. > 30% future liver remnant is preferred in fatty liver.
C. > 40% future liver remnant is recommended for severe fibrosis or cirrhotic liver.
D. ICG clearance is helpful for cirrhotic livers.
E. **All of the above.**

60. Gilbert’s disease:

A. Is a hyperbilirubinemia with serum T bili usually > 5
B. Is a progressive disease that usually leads to liver failure
C. Requires treatment with plasmapheresis
D. **Is an autosomal recessive disease with mildly elevated levels of unconjugated bilirubin and normally no serious consequences**
E. All of the above

61–75. Match the letter to the correct liver segment: 1, 2, 3, 4A, 4B, 5, 6, 7, 8.

(A segment may be used more than once)
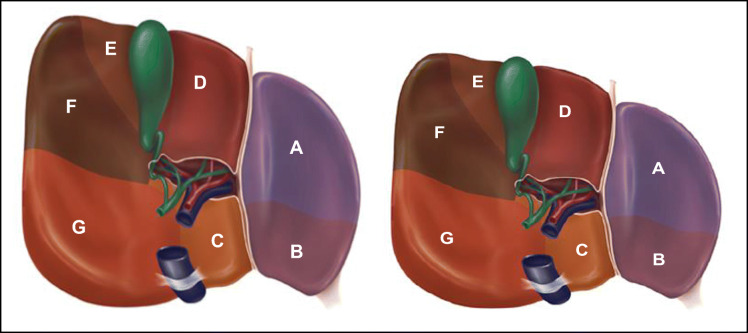


Segment #

61. A____**3**_______

62. B. ____**2**_______

63. C. ____**1**_______

64. D. ____**4B**______

65. E. ____**5**_______

66. F. ____**6**_______

67. G. ____**7**_______

68. H. ____**3**_______

69. I. _____**2**_______

70. J. ____**4B**_______

71. K. ____**4A**_______

72. L. _____**5**_______

73. M. ____**8**_______

74. N. _____**6**______

75. O. _____**7**______
